# Facteurs prédictifs de l’adhésion médicamenteuse chez les patients en insuffisance cardiaque chronique: expérience marocaine

**DOI:** 10.11604/pamj.2017.26.115.11471

**Published:** 2017-03-02

**Authors:** Yassine Ragbaoui, Imad Nouamou, Ayoub El Hammiri, Rachida Habbal

**Affiliations:** 1 Service de Cardiologie, Centre Hospitalier Universitaire IBN ROCHD, Casablanca, Maroc

**Keywords:** Adhésion médicamenteuse, insuffisance cardiaque, facteurs social et personnel, Medication adherence, heart failure, social and personal factors, prevalence

## Abstract

L’adhésion médicamenteuse chez les patients ayant une insuffisance cardiaque chronique est reconnue comme l’un des problèmes majeurs dans la gestion de cette pathologie. L’état démographique et socioéconomique des pays africains peut avoir un impact sur l’adhésion au traitement de l’insuffisance cardiaque chronique. Nous avons réalisé une étude transversale de Septembre 2014 à Janvier 2015 portant sur les patients en insuffisance cardiaque chronique suivis au centre d’insuffisance cardiaque du département de cardiologie du centre hospitalier universitaire IBN ROCHD à Casablanca au Maroc. La mesure de l’adhésion médicamenteuse était basée sur un questionnaire: questionnaire CARDIA. Les informations relatifs aux facteurs prédictifs d’adhésion médicamenteuse était dérivés du model d’adhésion multidimensionnel. Nous avons inclus dans cette étude 147 patients insuffisants cardiaques chroniques. Le pourcentage de l’adhésion médicamenteuse était de 83.6% selon CARDIA-Questionary. Les facteurs prédictifs qui influencent significativement l’adhésion médicamenteuse était: La dépression (p=0.034), le niveau de support social (p=0.03) et la prise de médicaments par le patient lui-même (p=0.0001). Comme dans plusieurs régions au monde, l’adhésion médicamenteuse chez les patients ayant une insuffisance cardiaque chronique reste un problème de santé au Maroc. Les différentes stratégies qui agissent sur les facteurs prédictifs pourraient améliorer l’adhésion médicamenteuse.

## Abstract (en)

Medication adherence in patients with chronic heart failure is recognized as one of the major issues in the management of this pathology. Demographic status and socioeconomic conditions in African countries may have an impact on chronic heart failure treatment adherence. We conducted a cross-sectional study of patients with heart failure treated in the center of heart failure in the Department of Cardiology at the IBN ROCHD University Hospital (Morocco) from September 2014 to January 2015. The extent of medication adherence was based on a questionary: CARDIA-Questionary. The informations related to predictive factors of medication adherence were derived from the multidimensional adherence model. 147 patients with chronic heart failure were included in the study. Medication adherence rate was 83.6%, according to CARDIA-Questionary. Predictive factors that significantly influenced medication adherence were: depression (p=0.034), level of social support (p=0.03) and patient self administration of the drugs (p=0.0001). Medication adherence in patients with chronic heart failure is a health problem in Morocco as well as in several world regions. Strategies affecting predictive factors might improve medication adherence.

## Introduction

L’adhésion médicamenteuse reste un problème clinique majeur dans la gestion des patients en insuffisance cardiaque chronique et il est reconnu que le manque d’adhésion médicamenteuse est un facteur de décompensation d’insuffisance cardiaque dans 40% des cas [1, 2]. Les interventions visant l’optimisation de l’adhésion du patient aux soins permettent de prévenir les hospitalisations et améliore la morbi-mortalité de l’insuffisance cardiaque [3]. Une connaissance des facteurs associés à l’adhésion médicamenteuse est nécessaire pour établir des interventions efficaces. Pour tenter de clarifier le rôle des différentes variables qui influent sur l’adhésion médicamenteuse un modèle d’adhésion multidimensionnel(MAM) a été proposé par l’organisation de l’organisation mondiale de santé, il est axé sur cinq dimensions: 1) au patient; 2) les facteurs liés à l’état clinique du patient; 3) facteurs liés au système des soins de santé et 4) les facteurs liés à des facteurs socioéconomiques. Le but de cette étude était d´évaluer l´adhésion au traitement médical et d’explorer les facteurs qui contribuent à l´observance du traitement médicamenteux chez les patients marocains atteints d´insuffisance cardiaque chronique en utilisant le modèle d´adhérence multidimensionnelle de l´Organisation mondiale de la santé.

## Méthodes

Nous avons mené une étude transversale entre Septembre 2014 et Janvier 2015 portant sur des patients en insuffisance cardiaque chronique suivis au centre d’insuffisance cardiaque du département de cardiologie du centre hospitalier universitaire IBN ROCHD à Casablanca au Maroc. Nous avons inclus les patients dont le diagnostic de l’insuffisance cardiaque était réalisé par un cardiologue, les patients étaient âgés de 18 ou plus. Les critères d’exclusion étaient la présence d’un déficit cognitif et l’incapacité de donner un consentement éclairé ou de participer à une entrevue. L’adhésion thérapeutique était définie par le degré de respect ou d´écart entre les prescriptions et les pratiques du patient en termes de santé. L’adhésion thérapeutique était mesurée par un questionnaire validé dans l’insuffisance cardiaque : CARDIA-questionnaire qui contenait trois questions: (1) Est-ce que le patient prenait son médicament presque tous le temps (prise de médicaments plus de 90%); (2) Est-ce que le patient prenait le médicament la plupart du temps (prise médicaments dans 75%-90% du temps); (3) Est-ce que le patient prenait le médicament moins de la moitié du temps [4]. Dans cette étude, on a recueilli les facteurs liés: (1) au patient: âge, sexe, les connaissances vis-à-vis des médicaments qui ont été apprécié par 3 questions: (1) est ce que le patient reconnait que son traitement est utilisé pour l’insuffisance cardiaque? (2) Est-ce que le patient reconnait le mécanisme de base de chaque médicament? (3) Est-ce que le patient sait le dosage quotidienne exact du traitement; on considérait les connaissances comme « optimales » si le patient répondait correctement à trois questions, «bonne» connaissance si deux réponses correctes, connaissance «suffisante» si une seule réponse correcte et connaissance «insuffisante» si aucune réponse n’est correcte. (2) l’état clinique du patient: sévérité des symptômes (NYHA), comorbidités, dépression apprécié par le questionnaire « Patient Health Questionnaire » (PHQ9). (3) la relation du patient au système de soins: apprécié par le questionnaire «Interpersonal Trust in a Physician Scale» [5]. Qui comporte 10 items notés sur une échelle de 5 (accord total) à 1(désaccord total). (4) Aux facteurs socioéconomiques: niveau d’éducation, la situation financière recueillie par un entretien avec le patient et la famille et l’aide social mesurée par l’échelle « Perceived Social Support Scale» [6] composé de 12 items. Toutes les analyses de données ont été réalisées avec le SPSS version 14. L’analyse des données a commencé avec un examen descriptif de toutes les variables, y compris les distributions de fréquences, les moyennes et les médianes. La différence était statistiquement significative pour P<0,05. Considérations éthiques: la participation à l´étude était volontaire. Le consentement était libre et éclairé, écrit ou verbal.

## Résultats

Nous avons inclus 147 patients insuffisants cardiaques chroniques. La moyenne d’âge était de 62ans+/-11. La cause la plus fréquente de l’insuffisance cardiaque chronique était la cardiopathie ischémique. L´échantillon était composé en grande partie de patients en stade II de la classification NYHA (61%), suivi du stade I (21%), stade III (17%) et enfin stade IV (1%).57% des patients avaient une (FE) entre 30-40%, 28% entre 40-50% et 15%<30%. Le taux de prévalence d’adhérence médicamenteuse était de 83%. L’adhésion thérapeutique était optimale (prise de médicaments dans 75% du temps ou plus) chez 122 patients, 120 patients prenait leurs médicaments presque tous le temps (prise de médicaments plus de 90%), 3 patients la plupart du temps (prise médicaments dans 75%-90% du temps), 24 patients moins de la moitié du temps. Les connaissances vis-à-vis des médicaments étaient insuffisantes chez 50% des patients, suffisantes dans 49% des cas et bonne dans 1% des cas. Chez le groupe des patients qui ne sont pas adhérents: la dépression était retrouvée chez 27, un niveau d’aide social faible chez 21 patients et chez 22 patients qui ne prenait pas le traitement par eux-mêmes. Après une analyse multivariée, nous avons trouvé que les facteurs prédictifs de mauvaise observance étaient: la dépression (p=0.034), le niveau de support social (p=0.03) et la prise de médicaments par le patient lui-même (p=0.0001).

## Discussion

D’après la littérature, c’est la première étude au Maroc dédiée à la recherche des facteurs influençant l’adhésion thérapeutique chez des patients en insuffisance cardiaque chronique. La mesure de l’adhérence médicamenteuse chez les patients en insuffisance cardiaque a un intérêt majeur parce qu’il a été prouvé que son amélioration permet d’améliorer le pronostic des patient [7]. La mesure d’adhérence médicamenteuse peut se faire par des méthodes subjectives basées sur les questionnaires ou bien par des moyens objectifs et dans certaines études dans la littérature les deux méthodes ont été utilisées. Le seuil de définition d’une mauvaise adhérence médicamenteuse diffère d’une étude à une autre[8], nous avons opté pour le seuil de 75% de prise médicamenteuse parceque nous avons trouvé qu’il était très souvent utilisé en littérature [7]. Dans cette étude nous n’avons pas trouvé de différence significative entre les deux sexes en termes d’adhérence médicamenteuse. Certaines études montrent que les femmes sont plus adhérentes que les hommes tandis que d’autres ont prouvé le contraire [9, 10]. L´un des principaux facteurs prédicteurs de l´adhésion dans cette étude a été le soutien social. Les patients qui ont reçu un soutien social adéquat des membres de leur famille et de leurs amis étaient plus adhérents. Les patients ont besoin d’un soutien émotionnel des membres de leur famille pour les aider à prendre leurs médicaments [11]. Sans assez de soutien social, il est difficile pour ce groupe de patients d´adhérer à la thérapeutique.

L’insuffisance cardiaque intéresse généralement une population âgée qui présente souvent des problèmes tels que l’oubli et la compréhension de la prise médicamenteuse, c’est ainsi qu’un soutien social peut prévenir ces obstacles à l’adhérence médicamenteuse. Les patients ayant l’habitude de prendre leurs traitements sans l’aide d’une tierce personne étaient plus adhérents que les patients dépendant d’autres personnes. Une méta-analyse comportant 31 études (18 245 patients) a examiné la relation entre la dépression et l’adhérence médicamenteuse dans un contexte de maladies cardiovasculaires et de diabète. Les auteurs de cette méta-analyse ont trouvé que les patients déprimés sont de 1.76 à 3.03 fois moins adhérents que les patients non déprimés [12]. Nous avons constaté aussi que les patients déprimés étaient moins adhérents que les autres, ces résultats concordent avec celle de Hansen et al. (2009) qui ont montré que la dépression était associée à une plus faible adhésion au traitement auto-rapportés chez des patients en insuffisance cardiaque [13].

Néanmoins notre étude présente quelques limitations : le nombre des patients étudiés était relativement réduit limitant ainsi la détection d’autres potentiels facteurs prédicteurs. La mesure de l’adhérence était basée sur un questionnaire or dans certaines études il a été montré qu’il existe une faible corrélation entre la méthode subjective et objective et qu’il est préférable d’associer les deux méthodes [14]. Notre étude montre l’intérêt d’une approche psycho-sociale dans la prise en charge d’un patient en insuffisance cardiaque chronique afin d’améliorer l’adhérence médicamenteuse. Des études seront nécessaires pour déterminer quelles seraient les méthodes les moins onéreuses et efficaces pour prendre en charge sur le plan psychosocial un patient en insuffisance cardiaque afin d’améliorer l’adhérence médicamenteuse.

## Conclusion

L’adhésion médicamenteuse dans l’insuffisance cardiaque reste un problème de santé au Maroc. Des stratégies incluant un soutien social et psychologique pourraient améliorer cette adhésion médicamenteuse.

### Etat des connaissances actuelle sur le sujet

- Facteurs d’adhésion médicamenteuse dans l’insuffisance cardiaque dans les pays occidentaux;
- Le pronostic de l’insuffisance cardiaque est influencé par l’adhésion médicamenteus;
- Le pronostic défavorable de l’insuffisance cardiaque.

### Contribution de notre étude à la connaissance

- La première étude au Maroc qui traite l’adhésion médicamenteuse des patients en insuffisance cardiaque;
- Nécessité d’une prise en charge psychologique et d’un soutien social des patients en insuffisance cardiaque;
- Age jeune de nos patients insuffisants cardiaques.

## References

[1] Chin MH, Goldman L (1997). Factors contributing to the hospitalization of patients with congestive heart failure. Am J Public Health..

[2] Tsuyuki RT, McKelvie RS, Arnold JM, Avezum A, Barretto AC, Carvalho AC (2001). Acute precipitants of congestive heart failure exacerbations. Arch Intern Med..

[3] Rich MW, Beckham V, Wittenberg C, Leven CL, Freedland KE, Carney RM (1995). A multidisciplinary intervention to prevent the readmission of elderly patients with congestive heart failure. N Engl J Med..

[4] Muzzarelli S, Brunner-La Rocca H, Pfister O, Foglia P, Moschovitis G, Mombelli G (2010). Adherence to the medical regime in patients with heart failure. Eur J Heart Fail..

[5] Balkrishnan R, Dugan E, Camacho FT, Hall MA (2003). Trust and satisfaction with physicians, insurers, and the medical profession. Med Care..

[6] Canty-Mitchell J, Zimet GD (2000). Psychometric properties of the Multidimensional Scale of Perceived Social Support in urban adolescents. Am J Community Psychol..

[7] Wu JR, Moser DK, De Jong MJ, Rayens MK, Chung ML, Riegel B (2009). Defining an evidence-based cutpoint for medication adherence in heart failure. Am Heart J..

[8] Gwadry-Sridhar FH, Arnold JM, Zhang Y, Brown JE, Marchiori G, Guyatt G (2005). Pilot study to determine the impact of a multidisciplinary educational intervention in patients hospitalized with heart failure. Am Heart J..

[9] Monane M, Bohn RL, Gurwitz JH, Glynn RJ, Avorn J (1994). Noncompliance with congestive heart failure therapy in the elderly. Arch Intern Med..

[10] Struthers AD, Anderson G, Donnan PT, MacDonald T (2000). Social deprivation increases cardiac hospitalisations in chronic heart failure independent of disease severity and diuretic non-adherence. Heart..

[11] Simpson SH, Farris KB, Johnson JA, Tsuyuki RT (2000). Using focus groups to identify barriers to drug use in patients with congestive heart failure. Pharmacotherapy..

[12] DiMatteo MR, Lepper HS, Croghan TW (2000). Depression is a risk factor for noncompliance with medical treatment: meta-analysis of the effects of anxiety and depression on patient adherence. Arch Intern Med..

[13] Hansen RA, Dusetzina SB, Song L, Gaynes BN, Tu W, Murray MD (2009). Depression affects adherence measurement but not the effectiveness of an adherence intervention in heart failure patients. Journal of the American Pharmacists Association : JAPhA..

[14] Garber MC, Nau DP, Erickson SR, Aikens JE, Lawrence JB (2004). The concordance of self-report with other measures of medication adherence: a summary of the literature. Med Care..

